# Emerging *Aeromonas* Species Infections and Their Significance in Public Health

**DOI:** 10.1100/2012/625023

**Published:** 2012-06-04

**Authors:** Isoken H. Igbinosa, Ehimario U. Igumbor, Farhad Aghdasi, Mvuyo Tom, Anthony I. Okoh

**Affiliations:** ^1^Applied and Environmental Microbiology Research Group (AEMREG), Department of Biochemistry and Microbiology, University of Fort Hare, Private Bag X1314, Alice 5700, South Africa; ^2^School of Public Health, University of the Western Cape, Bellville 7535, Cape Town, South Africa; ^3^Risk and Vulnerability Assessment Centre, University of Fort Hare, Private Bag X1314, Alice 5700, South Africa

## Abstract

*Aeromonas* species are ubiquitous bacteria in terrestrial and aquatic milieus. They are becoming renowned as enteric pathogens of serious public health concern as they acquire a number of virulence determinants that are linked with human diseases, such as gastroenteritis, soft-tissue, muscle infections, septicemia, and skin diseases. Proper sanitary procedures are essential in the prevention of the spread of *Aeromonas* infections. Oral fluid electrolyte substitution is employed in the prevention of dehydration, and broad-spectrum antibiotics are used in severe *Aeromonas* outbreaks. This review presents an overview of emerging *Aeromonas* infections and proposes the need for actions necessary for establishing adequate prevention measures against the infections.

## 1. Introduction

The aeromonads are Gram-negative, rod-shaped, facultative anaerobic, nonspore forming bacteria that are autochthonous and widely distributed in aquatic environments [1]. The genus is made up of psychrophiles and mesophiles from soil and aquatic environments and causes different kinds of diseases to many warm and cold-blooded animals. Several reconsiderations on the taxonomy and nomenclature of *Aeromonas* genus have been carried out over the years [2–4]. Although *Aeromonas* was initially positioned in the family Vibrionaceae, successive phylogenetic analyses point out that the genus *Aeromonas* is not closely related to vibrios resulting in the relocation of *Aeromonas* from the family Vibrionaceae to a new family, the Aeromonadaceae [2, 5]. The aeromonads and Enterobacteriaceae share many biochemical characteristics but are easily differentiated by oxidase test for which the aeromonads are positive. Generally, members of the genus are characteristically divided into three biochemically differentiated groups (*Aeromonas hydrophila*, *Aeromonas caviae,* and *Aeromonas sobria*), and these contain a number of genomospecies, and recently, new species have been added [3, 6]. Currently, the genus comprises of 17 DNA hybridization groups (HGs) or genomospecies and 14 phenospecies [7].

*Aeromonas *species are known as causative agents of a wide spectrum of diseases in man and animals [8]. Some studies have shown that some motile *Aeromonas *species are becoming food and waterborne pathogens of increasing importance [9, 10]. They have been associated with several food-borne outbreaks and are progressively being isolated from patients with traveler's diarrhea [11]. Presently, as a putatively emerging enteric pathogen, *Aeromonas *species have the inherent capability to grow in water distribution systems, especially in biofilms, where they may be resistant to chlorination [12]. Also, *A. hydrophila *is listed in the Contaminant Candidate List, and Environmental Protection Agency Method 1605 has validated its detection and enumeration in drinking water system [13].

In 1968, von Graevenitz and Mensch reviewed 30 cases of *Aeromonas *infections or colonization, which created awareness on their recognition as human pathogens and suggested that some aeromonads may be associated with gastrointestinal diseases. *Aeromonas *species are commonly isolated from fecal sample of children under the age of five years, whereas their isolation from other body sites usually occurred in adult populations. Aeromonads are known to cause severe diarrheal disease of short duration or chronic loose stools in children, the elderly, or the immuno-compromised individuals [1], and they have been implicated in travelers' diarrhea. The bacteria also cause cellulitis or wound infections due of traumatic injury in aqueous environment [14]. They also cause septicemia related to underlying diseases such as leukemia, cancer, cirrhosis, and various infections such as urinary tract infections, surgical wound infections, meningitis, peritonitis, and endocarditis [15]. Some predisposing factors for *Aeromonas *infection include hepatic diseases, diabetes, hematologic malignancies, hepatobiliary, and renal diseases [15].

Environmental source of *Aeromonas* pathogen involved in gastrointestinal infection was first reported by Holmberg and coworkers [16]. It was presented as an epidemiological study to backup the significance of untreated well water as a source of infection in patients with diarrheal disease. Phenotypic identification was relied upon before 1990s for the identification of *Aeromonas*, while several studies published thereafter identified isolates to hybridization groups. Hybridization groups containing virulence factors may be found in environmental and foods samples, but aeromonads will only cause gastroenteritis when their presence goes beyond an infective dose for a vulnerable host [17, 18]. In this paper, we present a general overview of *Aeromonas* species in the light of the increasing report of this group of bacteria as emerging pathogens and their effect on public health.

## 2. Taxonomy and Classification

Aeromonads were divided into two major groups based on physiological properties and host range until the late 1970s. Motile aeromonads grow at optimum temperature of 35–37°C and those predicted to cause human infections were recognized to be *A. hydrophila*. Nonmotile aeromonad which grows at 22–28°C and causes infections in fishes is called *Aeromonas salmonicida *[14]. Phenotypic markers for their differentiation include optimum growth temperature, motility, production of indole, and elaboration of a melanin-like pigment on tyrosine agar [7, 14]. Thereafter, the genus *Aeromonas *has advanced with the addition of new species and the reclassification of preexisting taxa [19]. In the past, *Aeromonas *species were placed alongside *Vibrio *species and *Plesiomonas shigelloides *in the family *Vibrionaceae*. However, genetic studies provide enough facts to support the placement of aeromonads in a family of their own called *Aeromonadaceae *[2].

The current classification of the genus *Aeromonas *is based on DNA-DNA hybridization and 16S ribosomal DNA relatedness, and the genera of the family *Aeromonadaceae *now include *Oceanimonas*, *Aeromonas*, *Tolumonas *(*incertae sedis*), and* Oceanisphaera*, [7]. The existing genomospecies and phenospecies within the genus *Aeromonas *are as listed in Table 1.

## 3. *Aeromonas* Species

The genus *Aeromonas *are made up of straight, coccobacillary-to-bacillary Gram-negative bacteria with surrounding ends measuring 0.3–1.0 × 1.0–3.5 *μ*m [7]. Most motile strains produce a single polar flagellum, while peritrichous or lateral flagella may be formed on solid media in some species [27]. *Aeromonas *species are facultative anaerobic, catalase, and oxidase positive, and chemoorganotrophic. They produce diverse kinds of extracellular hydrolytic enzymes such as arylamidases, esterases, amylase, elastase, deoxyribonuclease, chitinase, peptidases, and lipase [14, 19] and grow optimally at temperature ranges of between 22°C and 35°C, but growth can also occur at 0–45°C in a few species [8]. Some species, such as *A. salmonicida *strains, do not grow at 35°C [7]. All *Aeromonas *resist pH ranges from 4.5 to 9 but the optimum pH range is 5.5 to 9 and optimum sodium chloride concentration range is 0 to 4% [28].

*Aeromonas*' resistance to vibrostatic compound O/129 (150 *μ*g) and variable presence of ornithine decarboxylase activities differentiates the genus from *Plesiomonas *and *Vibrio* [14]. Other important distinguishing qualities include their inability to grow in the presence of 6.5% sodium chloride; ability to liquefy gelatin; inability to ferment i-inositol; negative string test. Some phenotypic characteristics include an inability to grow on thiosulfate citrate bile salts sucrose agar, and ability of most but not all *Aeromonas *species to ferment D-mannitol and sucrose [27]. The biochemical characteristics of *Aeromonas *species are as shown in Table 2.

A number of aeromonads are pathogenic for humans, and most human clinical isolates belong to hybridization groups (HGs) HG-1, HG-4, HG-8, HG-9, HG-10, HG-12, or HG-14 [14, 29]. The proportion of strains within these hybridization groups (HGs) that are capable of causing human disease is not well recognized. HG-2, HG-3, HG-5, HG-6, HG-7, HG-11, HG-15, HG-16, and HG-17 are isolated from the terrestrial or aquatic environment or from unhealthy animals, and they are not regarded as human pathogens [29]. Those capable of causing diseases in human are associated with a variety of infections including septicemia, meningitis, wound infections, peritonitis, and hepatobiliary infections [6].

## 4. Occurrence of *Aeromonas* Species

*Aeromonas *species are found globally in surface water, ground water, chlorinated drinking water, nonchlorinated drinking water, bottled mineral water [5, 12, 30], and broad range of foods [31]. They are found in the intestinal tract of humans and animals, raw sewage, sewage effluents, sewage-contaminated waters, and activated sludge [32, 33].

### 4.1. Occurrence of *Aeromonas* in Aquatic Environment

*Aeromonas *species have been found in chlorinated drinking water supplies in several countries [34, 35], and typically of densities less than 10 cfu/mL in drinking water distribution systems. They occur in distribution system biofilms where they may be protected from disinfection [12], and multiple strains are frequently found in water sources [36]. In the Netherlands, a drinking water standard of 200 cfu/100 mL at 25°C has been established for *Aeromonas* [37].

Maalej and colleagues [38] studied seasonal distribution of *Aeromonas *populations in urban effluent and natural seawater. They found 1.48 × 10^5^ to 2.2 × 10^8^ cfu/100 mL in the effluent, while seawater had counts lower than the detection limit to 7.9 × 10^3^ cfu/100 mL, and the seasonal abundance of *Aeromonas* was inversely related to the seasonal density of fecal coliforms.

Bonadonna et al. [39] studied the incidence of bacteria of anthropomorphic origin and those of autochthonous origin using model systems for prediction of public health risk to marine bathers. The resulting model used salinity, total coliforms, fecal coliforms, *Escherichia coli*, and location as analytical variables for presence of aeromonads. In the study, the presence of *Escherichia coli *and fecal coliforms were associated with lower *Aeromonas *counts, while prevalence of total coliform was associated with higher *Aeromonas *densities. Fecal coliforms and high salinity was associated with higher *Aeromonas *population. The complexity of the relationship between anthropomorphic and autochthonous bacteria confounds development of a predictive models for estimating public health risk of recreational exposure to marine waters.

### 4.2. Occurrence of *Aeromonas* in Food

Aeromonads have been isolated from food animals like fish, shellfish, meats, dairy products, and fresh vegetables. However, only few food borne outbreaks have been documented [28]. In a survey of all foods of animal origin carried out in India, aeromonads was isolated from fish (22%), snails (6.25%), and quail eggs (18%), buffalo milk (2.8%), and goat meat 8.9% [40]. These findings were in agreement with those of Tsai and Chen [41], who reported aeromonads in 22.2% of fish samples. Abbey and Etang [42] reported isolation of aeromonads in 28-29% of snails in Nigeria. Igbinosa and colleagues [43] also reported incidence of *Aeromonas* from some food samples including vegetable samples (35%), fresh fish (67%), smoked fish (70%), shrimps (60%), poultry (80%), meat (54%), meat products (80%), and raw milk (85%) in Benin City, Nigeria. Neyts et al. [18] reported the presence of *Aeromonas *species in densities of <2log_10_ to >5log_10_ cfu/g in 26% of vegetable samples, 70% of meat and poultry samples, and 72% of fish and shrimp samples in 68 food samples.

### 4.3. Occurrence of *Aeromonas* in Animal

The isolation of *Aeromonas* species from diseased fish, turtles, alligators, snakes, and frogs was the first implication of *Aeromonas* as animal pathogens [44]. *A. salmonicida* and *A. hydrophila* cause furunculosis, ulcerative disease, hemorrhagic disease, red sore disease, and septicemia in fish [45]. Lehane and Rawlin [46] investigated zoonoses acquired from fish and reported that aeromonads caused cellulitis, myositis, and septicemia as a result of injuries from handling fish, working in aquaculture, or keeping fish as pets. Also, *Aeromonas* species have long been recognized as pathogens of amphibians and reptiles [47].

*A. hydrophila* have been isolated from feces of normal horses (6.4%), pigs (9.6%), sheep (9.0%), and cows (21.1%) [48]. The total fecal carriage rate in animals is slightly higher than that in normal humans, which is <1 to 7% for most studies, although some studies report higher rates [49]. Figura and Marri [50] isolated *Aeromonas* species from the feces of domestic animals and found higher frequency of *A. hydrophila* than *A. caviae*. Stern et al. [51] isolated aeromonads from 3 of 21 turkeys and 1 of 32 cows, but none was detected from 22 pigs and 24 sheep. Gray and Stickler [52] reported findings of *A. hydrophila* predominantly in cow feces and *A. caviae* in pig feces. Also, Hathcock et al. [53] reported the isolation of *Aeromonas* species from feces, bedding, and drinking water of healthy cows and pigs.

Foods of animal origin including contaminated animals may play significant roles in the transmission of the aeromonads from food or animals to humans and animal feces appear to be the major source of contamination of foods. Aeromonads have recurrently been isolated from meat and edible organs of sheep and poultry, fish and seafood, raw milk, red meats, as well as pork and beef [54]. *A. hydrophila* was also isolated from diseased cyprinid loach in Korea [55]. The presence of aeromonads in food of animal origin may pose a public health problem for humans who come in contact with such animals.

### 4.4. Occurrence of *Aeromonas* in Human

Humans carry *Aeromonas *species in their gastrointestinal tract both in symptomatic and asymptomatic individuals. The rates of faecal carriage in persons in the absence of disease in developed countries range from 0% to 4% [56], while the isolation rate from persons with diarrheal illness ranges from 0.8 to 7.4% [57].

Sinha et al. [58] reported aeromonads in 6.5% of all patients in India, while in Hong Kong, Chan et al. [59] documented aeromonads in 6.9% of adult patients with acute diarrhoea. Others reported isolation rates from symptomatic patients from 0.04% to 21% [60, 61]. In Limpopo Province of South Africa, Obi and Bessong [62] reported the isolation of *Aeromonas *sp. from 13.3% HIV patients with chronic diarrhoea in rural communities. Immunocompromised people can also suffer from *Aeromonas*-associated chronic diarrhoea. In Saudi Arabia, Ibrahim and Colleagues [63] reported two cases of chronic colitis from immunocompromised patients associated with *A. hydrophila*.

## 5. Pathogenesis and Virulence Factors

*Aeromonas *species produces a broad range of extracellular enzymes, some of which are thought to contribute to pathogenesis. Virulence of aeromonads depends on several factors and also incompletely understood despite decades of intense research [64]. *Aeromonas *is said to be pathogenic because it possess all the requirements of pathogenic bacteria. It attaches and enters into host cells through production of flagella, pili and adhesins [65]. Multiplication in host tissue is assisted by the production of siderophores and outer membrane proteins, while production of capsule, S-layer, lipopolysaccharide, and porins contributes to their resistance to host defenses mechanism. Enterotoxins, proteases, phospholipases, and hemolysins cause damage to host cells leading to cell death [65]. Several extracellular products are elaborated, including cytotoxic and cytotonic enterotoxins, hemolysins, and various hydrolytic enzymes. The occurrence of both Type II and Type III secretions systems has been demonstrated [66], including the presence of several virulence factors such as enolase.

Some aeromonads produce a range of cell surface and secretory proteases that may augment their virulence [67]. It is well documented that a significant proportion of the *A. hydrophila *isolated from chlorinated and nonchlorinated water contained genes responsible for enterotoxigenic or cytotoxic activity [68]. Also, environmental temperature has been reported to be a parameter in the expression of virulence factors. *A. hydrophila* isolated from the environment produced significantly less enterotoxins when grown at 37°C compared to 28°C, while the clinical isolates examined produced more enterotoxins at 37°C than at 28°C [69]. The temperature of the human body is approximately 37°C; therefore, strains that produce virulence factors at this temperature are likely to be more significant as human pathogens.

## 6. Clinical Infections

Clinical indications of *Aeromonas* infection usually depend upon the site and severity of infection [70]. Wound infections often result in cellulitis and rarely necrotizing fasciitis. Septicemia may accompany wound infection or may be secondary to systemic diseases such as cirrhosis, cancer, biliary disease, diabetes, or diseases resulting in gastrointestinal perforations [71], and dissemination may give rise to meningitis or endocarditis. Pneumonia is infrequent and it is usually associated with aspiration, such as in near drowning [72]. Gastroenteritis symptoms vary from mild self-limiting to dysentery or cholera-like illness. Both gastrointestinal and extraintestinal infections are now known to occur in previously healthy hosts as well as immunocompromised or, otherwise, susceptible individuals [15].

### 6.1. Gastrointestinal Infection

*Aeromonas* species have increased in recent years since they cause gastroenteritis, cellulitis, peritonitis, meningitis, and pneumonia in immunocompromised humans and disseminated infections in immunocompromised hosts [11]. In developing countries, *A. hydrophila* is also known to cause diarrhea in children [73] and travelers [74]. Known risk factors that predispose humans to the disease include drinking or swimming in contaminated water and also ingestion of contaminated food.

Though *Aeromonas *species have been recognized as enteric pathogens, their mechanisms of pathogenicity remain vague. *A. caviae *bind to mucosal epithelial cells [35], and the majority of aeromonads related to gastroenteritis include *A. veronii *biovar sobria (HG-8/10), *A. hydrophila *(HG-1), and *A. caviae *(HG-4), though *A. veronii *biovar veronii (HG-8/10), *A. trota *(HG-13), and *A. jandaei *(HG-9) occur rarely [11]. Gastroenteritis attributed to *A. sobria *was characterized by acute watery, abdominal pain, vomiting, diarrhea, and fever [70]. Goldsweig and Pacheco [75] also reported on infectious colitis caused by *Aeromonas *species.

### 6.2. ExtraIntestinal Infection

Extraintestinal infections of environmental origins arise directly from soil or water contact, or indirectly by ingestion and bacteremic dissemination of aeromonads from the gastrointestinal tract [11]. The two major routes of infection are environment-water-animals complex and ingestion of contaminated foods [71].

In immunocompromised persons, bone infections appear to be a result of blood-borne spread [15]. In healthy persons, bone infection is next to tissue trauma, often arising after contamination in freshwater [14]. Invasive infections at other sites occur primarily in immunocompromised patients, particularly those with leukemia, malignancy, cirrhosis of the liver, or immunosuppression cases [15]. In cases of *Aeromonas* septicemia different from those of soft-tissue infections, the origin of the organism and portal of entry is yet unclear. An endogenous source has been proposed as the locus of infection from which subsequent bloodstream invasion arises [72].

## 7. Epidemiology and Disease Outbreak in *Aeromonas*

Aeromonads are commonly isolated from drinking water [12, 30], and temporal and seasonal relationship between presence of aeromonads in drinking water and their presence in the stools of patients with gastroenteritis have been reported [11]. Yamada et al. [76] reported incidence of *Aeromonas *infections in Japanese travelers to developing countries. *Aeromonas *species have been reported as the cause of diarrhea in 2% of travelers to Africa, Latin America and Asia [77, 78]. Human carriage of *Aeromonas *species could be symptomatic or asymptomatic. In developed countries, the rates of fecal carriage in asymptomatic persons range from 0% to 4% [34], while the isolation rate from individuals with diarrheal illness ranges from 0.8 to 7.4% [35]. The rate of recovery among children with diarrhea vary geographically: 0.62 to 4% was reported in Malaysia [79]; 2% in Sweden [34]; 0.75% in Nigeria [80]; 4.8% in Switzerland [81]; 2.3% in Taiwan [82]; and 6.8% in Greece [83]. In India, aeromonads was reported in 6.5% of all patients [36]. Chan et al. [37] also documented the incidence of aeromonads in 6.9% of adult patients with acute diarrhea in Hong Kong. Previous reports on incidence rates from symptomatic patients range from 0.04% to 21% [38, 39]. Generally, the isolation rates in human fecal specimens vary as geographical areas, food habits, level of sanitation, patient populations, and isolation methods affects the recovery rates [38].

## 8. Diagnosis and Detection of *Aeromonas* Species

To detect outbreaks efficiently, public health surveillance, and diagnostic procedures for *Aeromonas* species require both sensitivity and specificity. The approaches of Abbott et al. [4] and Carnahan and Joseph [7] make it possible to identify nearly every isolate to species levels. 

### 8.1. Culture-Based Detection Methods

Aeromonads grow on isolation media, and a huge number of selective and differential isolation media have been developed for the recovery of *Aeromonas *species from the environment, foods, and clinical specimens [84]. Comparative study suggest that no single medium results in optimum recovery of aeromonads, and combinations of different isolation media and methods are frequently employed by direct plating, membrane filtration, or multiple tube tests for determining most probable numbers. The membrane filtration method, EPA Method 1605, has been authenticated for the isolation of *A. hydrophila *from drinking water samples [13].

Several culture enrichment and culture media have been evaluated for the recovery of aeromomads from foods [31]. Starch ampicillin agar (SAA) and bile salts inositol brilliant green agar (BIBG) with initial enrichment in alkaline peptone water (APW) or tryptose broth containing ampicillin (TSB-30, ampicillin 30 mg/L) are recommended simultaneously with commercially available media such as Aeromonas Medium (Ryan's Medium). Starch glutamate ampicillin penicillin (SGAP-10) medium was used in the isolation of aeromonads from sewage sludge [85]. This medium is highly selective, and it has been used to detect aeromonads from foods and other samples matrices. *Aeromonas *species grow readily in blood culture media and on 5% sheep blood agar used in clinical laboratories for detection and isolation of human pathogens from normally sterile body sites [31]. Isolation of aeromonads from contaminated samples such as feces require the use of selective and differential media such as McConkey agar, cefsulodin irgasan novobiocin (CIN) agar, or blood ampicillin agar (10 mg/L ampicillin). To facilitate recovery of aeromonads from highly contaminated samples such as feces, enrichment broths such as alkaline peptone water are incubated overnight and subcultured onto CIN agar and blood ampicillin agar [85]. Culture plates are then incubated aerobically at 35°C for 24–48 h. *Aeromonas *species produce distinctive colonies, with or without hemolysis on blood agar. Colonies are screened by carrying out oxidase test and identified using biochemical methods or commercially-available bacterial identification kits [13, 85].

### 8.2. Molecular Detection and Identification Methods

Polymerase chain reaction (PCR) methods have been developed to detect the presence of *Aeromonas *species in a wide range of samples. Özbas et al. [86] developed a PCR method for the detection of *A. hydrophila *in raw milk. The detection limit was 2log_10_ cfu/g and the detection rate was 23% for PCR method and 14% for culture method. Stine et al. [87] constructed a microarray of DNA probes to study the population dynamics of microbial communities and used the microarray to study population dynamics and interactions of marine bacteria in coastal waters where aeromonads were found to make up a large proportion of the microbial flora. Galindo and coworkers [88, 89] also used microarrays to detect *A. hydrophila *cytotoxic enterotoxin-inducing genes in macrophages, thus revealing the potential of microarrays in elucidating intracellular mechanisms of pathogenesis. Galindo et al. [90] used microarrays and proteomics to examine the effects of cytotoxic enterotoxin on human epithelial cells.

Some researchers have developed probes for the detection of various *Aeromonas *species [91, 92]. Jun et al. [55] employed the restriction fragment length polymorphism (RFLP) method to distinguish *A. salmonicida *and *A. bestiarum.* The genetic heterogeneity of aeromonads as the result of crossover in ribosomal sequences makes it unlikely that 16S ribosomal DNA will be a practical means of differentiation of species [93], since additional endonucleases must be added as new species are recognized, resulting in an unwieldy and overly complex typing system [94, 95].

The 16S-23S intergenic spacer regions were sequenced, and it was found that the resulting phylogeny did not agree with the results of 16S ribosomal DNA and DNA-DNA hybridization studies [96]. The sequence analysis of the *gyrB *gene was used to construct a phylogenetic tree of all 17 hybridization groups [97] and *Aeromonas culicicola *was grouped with *Aeromonas veronii*, but it grouped with *Aeromonas jandaei *based upon 16S ribosomal gene sequence. The sequence analysis of the polymerase chain reaction amplicons of the *gyrB *gene was viewed as a better phylogenetic chronometer than the 16S ribosomal gene. Yanez et al. [98] have documented that the *gyrB *gene agree with the 16S ribosomal data which led to placement of the genus *Aeromonas *in the family *Aeromonadaceae*, and *gyrB *gene sequences were useful in resolving discrepancies between 16S ribosomal gene sequences and DNA-DNA hybridization results.

Minana-Galbis et al. [99] also examined the genetic diversity among *A. hydrophila, A. bestiarum, A. salmonicida *and *Aeromonas popoffii *by multilocus enzyme electrophoresis (MLEE). *Aeromonas popoffii *and *A. bestiarum *were found to be closely related this method. MLEE has been used in genomospecies determination since 1991 [100]. Multilocus sequence typing (MLST) using the four gene loci of 16S rDNA, *recA*, *gyrB, *and* chiA *has revealed the taxonomic limitations of 16S rDNA alone [101].

## 9. Prevention and Treatment

As highlighted previously, aeromonads are ubiquitous in many environmental waters. As a result, they are present in most source waters used for drinking water production. The existing techniques used for treatment and disinfection are effective in minimizing the level of aeromonads in the finished drinking water. It has been reported that *A. hydrophila* is usually more susceptible to chlorine and monochloramine than coliforms [12]. The most efficient approach for controlling *Aeromonas* growth is to limit the *Aeromonas* species entering the distribution system through effective treatment and maintenance, to maintain temperatures below 14°C, to provide free-chlorine residuals above 0.1–0.2 mg/L, and to limit the levels of organic carbon compounds in the water [102]. However, it is difficult to manage its growth in biofilms [12, 102].

Rehydration therapy is adequate intervention in most pediatric cases of gastroenteritis and watery diarrhea caused by *Aeromonas *species [102]. Draining obstructions and antibiotic therapy are effective in the management of infection in patients with acute suppurative cholangitis [103]. Surgical intervention might be necessary in cases of necrotizing fasciitis. In addition, cellutis may require debridement, and abscesses may require draining [103].

Gastrointestinal infections caused by aeromonads are generally self-limiting, and antibiotic therapy is required only in prolonged cases in immunocompromised hosts. Antimicrobials are employed for only severe and unresponsive cases of *Aeromonas *gastroenteritis [103] or extraintestinal infections [71, 73].

*Aeromonas *species demonstrate differences in their susceptibilities to antibiotics. Clinical isolates of *A. caviae *are more susceptible to ticarcillin than *A. veronii *and *A. hydrophila. *Use of clavulanic acid with amoxicillin enhances antibacterial activity but tazobactam did not enhance the effect of piperacillin and *A. veronii *(79%) showed higher susceptibility to cefazolin than *A. caviae *(53%) or *A. hydrophila *(40%) [104].

Ko et al. [105] have established that minocycline and cefotaxime administered simultaneously produced a synergistic effect against *A. hydrophila *using a murine model. Also, ciprofloxacin was as effective as cefotaxime-minocycline *in vitro* and in a murine model, suggesting that clinical studies are necessary [106].

## 10. Antimicrobial Resistance

The origins of antibiotic resistance in the environment is relevant to human health because of the increasing importance of zoonotic diseases as well as the need for predicting emerging resistant pathogenic organisms [107]. Antibiotic-susceptibility pattern is also significant for selective isolation of microorganisms. The aeromonads have been regarded universally to exhibit resistance to the penicillins (penicillin, ampicillin, carbenecillin, and ticarcillin) for quite a long time [108]. In addition to selection of antibiotic therapy in the clinical setting, antibiotic sensitivity patterns are sometimes useful as phenotypic characteristics for species identification, especially for clinical isolates [108]. Most of *Aeromonas* species show susceptibilities to aminoglycosides, tetracycline, chloramphenicol, trimethoprim-sulfamethoxazole, and quinolones [108]. They are also susceptible to piperacillin, azlocillin, and the second and third generation of cephalosporins [108]. Sen and Rodgers [36] reported antibiotic susceptibility tests on 164 strains of *Aeromonas*, and resistance to nalidixic acid (54–62%), ciprofloxacin (12–22%), and norfloxacin (14–19%) were observed.

In the United States, over 90% of aeromonad strains were susceptible to third-generation cephalosporins and aminoglycosides and almost all the aeromonads were susceptible to quinolones [109]. Also, in the United States, most strains were susceptible to chloramphenicol, tetracycline, minocycline, doxycycline, and nitrofurantoin, but resistant to vancomycin, clindamycin, and erythromycin. Imipenem was reported to be efficient for treatment of *Aeromonas *infections [110]. Petersen and Dalsgaard [111] found that most of their *Aeromonas* strains were resistant to the commonly used antibiotics such as chloramphenicol, tetracycline, and trimethoprim. Huys et al. [112] found oxytetracycline-resistant *Aeromonas* strains in water from fish farms and in hospital sewage.

The use of antibiotics in aquaculture plays an important role in the amplification of resistance in a given reservoir. Multiple antibiotic resistances (MARs) between *Aeromonas* species have been reported globally by different authors [36, 113, 114]. Radu et al. [115] reported the frequent occurrence of multiple antimicrobial resistances and the presence of similar resistance patterns in some *A. hydrophila*, *A. veronii* biovar *sobria*, and *A. caviae* strains isolated from fish. Most of the *A. salmonicida* strains isolated by Kirkan and colleagues [116] were resistant to erythromycin, amoxycillin+clavulanic acid, penicillin, gentamicin, oxytetracycline cefuroxime sodium, and sulphamethoxazole+trimethoprim. *A. hydrophila* isolated from fish samples in Mhow city, India, showed 100% sensitivity to ciprofloxacin, cefuroxime, ceftriaxone, cephotaxime, chloramphenicol, gentamycin, kanamycin, nitrofurantoin, nalidixic acid, and ofloxacin, while 62.2% and 50% of the bacteria were susceptible to cotrimoxazole and oxytetracycline, respectively. On the other hand, all isolates were resistant to colistin and ampicillin [113].

Resistance to chloramphenicol is very rare in *Aeromonas *species. Michel et al. [117] reported that minimum inhibitory concentrations (MICs) of chloramphenicol against *A. salmonicida *strains displayed a bimodal distribution and demonstrated the existence of a large and well-delineated resistant population. Distribution of MIC values of chloramphenicol in *A. salmonicida *strains were 0.25–2 *μ*g/mL and 16–>256 *μ*g/mL, whereas in motile aeromonads it ranged from 0.06 *μ*g mL^−1^ to >256 *μ*g/mL. Conversely, Guz and Kozińska [118] findings revealed that *Aeromonas *species are susceptible to chloramphenicol (MIC >0.06–2 mg/L, MIC_90_ 0.5 mg/L). Chloramphenicol is hazardous to humans, it causes an idiosyncratic, aplastic anemia, and at present, it is extremely illegal to use in food animals.

Edwards and coworkers [119] documented antibiotic resistance in *Aeromonas *species isolated from a eutrophic lake in England. Goñi-Urriza and colleagues [32, 120] reported antibiotic resistance to trimethoprim (42%), pipemidic acid (67%), streptomycin (65%), cephalothin (93%), cefoxitin (56%), ticarcillin (87%), sulfamethoxazole (90%), naladixic acid (59%), ampicillin (99%), oxolinic acid (67%), and tetracycline (14%) in *Aeromonas *species isolated from European rivers. Susceptibility to fluoroquinolones differ from 54% to 98%. Most strains were susceptible to ciprofloxacin, fosfomycin, colistin, gentamicin, cotrimoxazole, cefotaxime, chloramphenicol, tobramycin, and imipenem [120], and urban wastewater effluents are thought to be a contributing factor to the increasing rate of antibiotic resistance in environmental aeromonads [120].

## 11. Public Health Control

The prevention of the spread of *Aeromonas* strains involved in diarrhea depends on ensuring appropriate sanitary measure like proper food preparation, hand-washing, and efficient sewage disposal system. Proper surveillance of water, food, and sanitation facilities, using public health diagnostic and detection procedures is essential to protect individuals including infants from *Aeromonas* strain diarrhea.

In the aquaculture environment, management consists of disposal of diseased animals and water treatment to control the densities of aeromonads in aquaculture systems. Maintaining high standards of water quality, temperature control, disinfection of equipment, and stress reduction reduce the prevalence of disease [102]. In aquaculture system the quality of the water is of paramount importance in the prevention of contamination of fish or plants grown in wastewater ponds. Reliance has been placed principally on minimizing the risk of pathogen transmission by thorough cooking of the products, but this approach has not always been satisfactory and, where the pond products are eaten uncooked, no health protection is provided. Therefore, the presence of potentially pathogenic and multidrug-resistant *Aeromonas *strains in surface waters remains of significant health implication. Continuous monitoring of surface waters is vital to identify potential water-borne pathogens and to reduce the health risk caused by the Aeromonads.

## 12. Conclusion

*Aeromonas* infection remains among those infectious diseases of potentially serious threat to public health. *Aeromonas* disease outbreaks have created a painful awareness of the personal, economic, societal, and public health costs associated with the impact of contaminated water in the aquatic milieu. There is evidence to suggest that the prevalence of *Aeromonas* infections may be dramatically underestimated in developing nations and that routine endemic exposure to waterborne and food-borne pathogens may occur more frequently than originally perceived. A variety of demographical, societal, environmental, and physiological emergence factors likely play critical roles in enhancing the frequency of transmission of pathogens to hosts and the increasing trend in antibiotics resistance in the bacteria makes extended studies on the bacteria imperative and this is subject of investigation in our group.

## Figures and Tables

**Table 1:** Hybridization groups (genomospecies) and phenospecies of the genus *Aeromonas*.

DNA hybridization group (HG)	Type strain/Reference	Genospecies	Phenospecies	Remarks
1*	ATCC 7966	*A. hydrophila *	*A. hydrophila *	Isolated from clinical specimens
1*	BCCM/LMG 19562	*A. hydrophila *subsp. *dhakensis *	*A. hydrophila *subsp. *dhakensis *	Isolated from clinical specimens
1*	BCCM/LMG 19707	*A. hydrophila *subsp. *ranae *	*A. hydrophila *subsp. *ranae *	Pathogenic for frogs
2*	ATCC 14715	*A. bestiarum *	*A. hydrophila*-like	Isolated from clinical specimens
3*	ATCC 33658	*A. salmonicida *	*A. salmonicida *subsp. *salmonicida *	Nonmotile fish pathogen
3*	ATCC 33659	*A. salmonicida *	*A. salmonicida *subsp. *achromogenes *	Nonmotile fish pathogen
3*	ATCC 27013	*A. salmonicida *	*A. salmonicida *subsp. *masoucida *	Nonmotile fish pathogen
3*	ATCC 49393	*A. salmonicida *	*A. salmonicida *subsp. *smithia *	Nonmotile fish pathogen
3*	CDC 0434-84, Popoff C316	unnamed	*A. hydrophila*-like	Isolated from clinical specimens
4*	ATCC 15468	*A. caviae *	*A. caviae *	Isolated from clinical specimens
5A*	CDC 0862-83	*A. media *	*A. caviae*-like	Isolated from clinical specimens
5B*	CDC 0435-84	*A. media *	*A. media *	
6*	ATCC 23309	*A. eucrenophila *	*A. eucrenophila *	
7*	CIP 7433, NCMB 12065	*A. sobria *	*A. sobria *	
8X*	CDC 0437-84	*A. veronii *	*A. sobria *	
8Y*	ATCC 9071	*A. veronii *	*A. veronii *biovar sobria	Isolated from clinical specimens
9*	ATCC 49568	*A. jandaei *	*A. jandaei *	Isolated from clinical specimens
10*	ATCC 35624	*A. veronii *biovar *veronii *	*A. veronii *biovar veronii	Isolated from clinical specimens, ornithine decarboxylase positive
11*	ATCC 35941	unnamed	*Aeromonas *spp. (ornithine positive)	
12*	ATCC 43700	*A. schubertii *	*A. schubertii *	Isolated from clinical specimens
13*	ATCC 43946	*Aeromonas *Group 501	*A. schubertii*-like	Isolated from clinical specimens
14*	ATCC 49657	*A. trota *	*A. trota *	Isolated from clinical
15*	ATCC 51208, CECT 4199	*A. allosaccharophila *	*A. allosaccharophila *	
16*	ATCC 51020,	*A. encheleia *	*A. encheleia *	Pathogenic for eels
17*	BCCM/LMG 1754	*A. popoffii *	*A. popoffii *	
Unassigned*	MTCC 3249, NCIM 5147	*A. culicicola *	*A. culicicola *	Isolated from mosquitoes
Unassigned	[20]	*A. eucrenophila*	*A. tecta*	Isolated from clinical and environmental sources
Unassigned	[21]	*A. trota*	*A. aquariorum*	Isolated from monkey faeces
Unassigned	[22]	*A. popoffii*	*A. bivalvium*	Isolated from aquaria of ornamental fish
Unassigned	[23]	unnamed	*A. sharmana*	Isolated from bivalve molluscs
Unassigned	[24]	*A. encheleia*	*A. molluscorum*	Isolated from bivalve molluscs
Unassigned	[25]	*A. schubertii*	*A. simiae*	Isolated from midgut of mosquitoes
Unassigned	[26]	*A. jandaei*	*A. culicicola*	Isolated from a warm spring

*Carnahan and Joseph [7].

**Table 2:** The Biochemical Identification of Motile *Aeromonas *species.

Characteristic	*A. hydrophila*	*A. bestarium*	*A. caviae*	*A. media*	*A. eucrenophila*	*A. sobria*	*A. veronii biovar sobria*	*A. jandaei*	*A. veroni biovar veroni*	*A. schubertii*	*A. trota*	*A. allosaccharophila*	*A. encheleia*	*A. popoffii*
Hybridization group	HG-1	HG-2	HG-4	HG-5	HG-6	HG-7	HG-8	HG-9	HG-10	HG-12	HG-14	HG-15	HG-16	HG-17
Esculin hydrolysis	+	+	+	+	**+**	**−**	**−**	**−**	**+**	−	**−**	d	**+**	**−**
Gas from glucose	**+**	**+**	**−**	**−**	**+**	**+**	**+**	**+**	**+**	**−**	**+**	**+**	**+**	**+**
Voges-Proskauer	**+**	**+**	**−**	**−**	**−**	**+ **weak	**+**	**+**	**+**	d	**−**	**−**	**−**	**+**
Indol	**+**	**+**	**+**	d	+	+	+	+	+	−	+	+	+	d
Pyrazinzmidase	**+**	**−**	**+**	d	+	nd	−	−	−	−	−	nd	nd	nd
L-arabinose	d	**+**	**+**	**+**	**+**	**−**	**−**	**−**	**−**	**−**	**−**	d	−	nd
D-mannitol	**+**	**+**	**+**	**+**	**+**	**+**	**+**	**+**	**+**	**−**	**+**	**+**	**+**	**+**
Sucrose	**+**	**+**	**+**	**+**	d	**+**	**+**	**−**	**+**	**−**	**−**	**+**	**+**	**−**
Lysine decarboxylase	**+**	**−**	**−**	**−**	**−**	+ weak	**+**	**+**	**+**	**+**	**+**	**+**	**−**	**−**
Ornithine decarboxylase	**−**	**−**	**−**	**−**	**−**	**−**	**−**	**−**	**+**	**−**	**−**	d	**−**	**−**
Arginine dihydrolase	**+**	**+**	**+**	**+**	**+**	**−**	**+**	**+**	**−**	**+**	**+**	d	d	**+**
Arbutin hydrolysis	**+**	**+**	**+**	**+**	**+**	nd	−	−	+	−	−	nd	−	nd
H_2_S production	**+**	**+**	**−**	**−**	**+**	nd	**+**	**+**	**+**	**−**	**+**	nd	nd	nd
Hemolysis	**+**	**+**	d	−	−	−	+	+	+	+	+	nd	nd	**−**
Ampicillin 10 *μ*g	R	R	R	nd	nd	S	R	R	R	R	S	nd	R	nd
Carbenicillin 30 *μ*g	R	R	R	nd	nd	S	R	R	R	R	S	nd	R	nd
Cephalothin 30 *μ*g	R	R	R	d	S	S	S	S	D	S	R	nd	nd	nd
Colistin 4 *μ*g/mL	d	d	S	S	S	nd	S	R	S	S	S	nd	nd	nd

HG: hybridization group;** +**:** >**75% of strains positive; d: 26–74% of strains positive; −: <25% of strains are positive; nd: not determined; R: resistant; S: sensitive.

Source**: **Carnahan and Joseph [7].
